# UPF1 promotes chemoresistance to oxaliplatin through regulation of TOP2A activity and maintenance of stemness in colorectal cancer

**DOI:** 10.1038/s41419-021-03798-2

**Published:** 2021-05-21

**Authors:** Congcong Zhu, Long Zhang, Senlin Zhao, Weixing Dai, Yun Xu, Yuqin Zhang, Hongtu Zheng, Weiqi Sheng, Ye Xu

**Affiliations:** 1 Department of Colorectal Surgery, Fudan University Shanghai Cancer Center, Shanghai, 200032 PR China; 2 Department of Oncology, Shanghai Medical College, Fudan University, Shanghai, 200032 PR China; 3 Department of Pathology, Fudan University Shanghai Cancer Center, Shanghai, 200032 PR China

**Keywords:** Prognostic markers, Oncogenes

## Abstract

UPF1 is proved to dysregulate in multiple tumors and influence carcinogenesis. However, the role of UPF1 in oxaliplatin resistance in colorectal cancer (CRC) remains unknown. In our study, UPF1 is upregulated in CRC in mRNA and protein levels and overexpression of UPF1 predicts a poor overall survival (OS) and recurrence-free survival (RFS) in CRC patients and is an independent risk factor for recurrence. UPF1 promotes chemoresistance to oxaliplatin in vitro and in vivo. UPF1-induced oxaliplatin resistance can be associated with interaction between zinc finger of UPF1 and Toprim of TOP2A and increasing phosphorylated TOP2A in a SMG1-dependent manner. Moreover, UPF1 maintains stemness in a TOP2A-dependent manner in CRC. Taken together, UPF1 was overexpressed and predicted a poor prognosis in CRC. UPF1 enhanced chemoresistance to oxaliplatin in CRC, which may result from regulation of TOP2A activity and maintenance of stemness. Our findings could provide a new therapy strategy for chemoresistance to oxaliplatin in CRC patients.

## Introduction

Colorectal cancer (CRC) is the third most frequent for incidence and the second most frequent for mortality worldwide^1^. However, in China, CRC is the fourth most common and the fifth leading cause of cancer death^2^. Surgical resection is the potential radical treatment for CRC. Other than surgery, radiochemotherapy, targeted therapy, and immunotherapy can be applied to CRC patients with lymph node metastases or distant metastases^3–5^. However, the acquisition of drug resistance is a major hurdle for good clinical prognosis^6^.

Oxaliplatin, a third-generation platinum coordination complex, can be used for treatment in multiple cancers^7–9^. Oxaliplatin-based combined chemotherapy is routinely applied in advanced and metastatic CRC and significantly increases the overall survival (OS) rate and metastases resection rate^10^. Unfortunately, acquired or intrinsic resistance brings failure to treatment. The underlying mechanisms of oxaliplatin resistance include DNA damage response and repair, inhibition of cell death, cellular transport, detoxification, and epigenetic alteration^11^.

UPF1, an mRNA surveillance factor, is a RNA-dependent ATPase and helicase for nonsense-mediated decay of mRNAs containing premature stop codons^12^. In addition, UPF1 is proved to dysregulate and influence carcinogenesis in multiple tumors. UPF1 is commonly mutated in pancreatic adenosquamous carcinoma and there is little or no UPF1 expression in many adenosquamous carcinoma tumors compared to adjacent normal tissue^13^. It is revealed that UPF1 is downregulated in hepatocellular carcinoma and inhibits the tumor progression^14,15^. However, the role of UPF1 in oxaliplatin resistance in CRC remains unclear.

Topoisomerases are enzymes responsible for overcoming topological problems in the process of DNA replication, transcription and repair, and proved therapeutic targets of anticancer^16^. Human topoisomerase II-α (TOP2A) plays an important role in organizing genome structure and promoting chromosome segregation^17^. Growing lines of evidence indicate that TOP2A is overexpressed in a variety of tumors, such as lung cancer, hepatocellular cancer, and colorectal cancer. Elevated expression of TOP2A is associated with advanced stage of disease, aggressive phenotype of tumor, and poor prognosis^18^. Targeting TOP2A is a promising approach in cancer therapy and some agents have been approved in clinical use, such as etoposide and teniposide^18^. As an enzyme, the activity of TOP2A can be regulated by protein–protein interactions and post-translational modifications (phosphorylation, ubiquitination, and SUMOylation)^19–21^.

In our study, we demonstrated that UPF1 was overexpressed in CRC tissues and high expression of UPF1 predicted a poor prognosis of CRC patients. UPF1 promoted oxaliplatin resistance both in vitro and in vivo in CRC. UPF1-induced oxaliplatin resistance can be associated with elevated phosphorylation of TOP2A and maintenance of stemness. These findings contribute to improving our understanding of oxaliplatin resistance, and may provide a new therapy strategy for chemoresistance to oxaliplatin in CRC patients.

## Results

### UPF1 is upregulated and predicts a poor prognosis in CRC

**Fig. 1:**
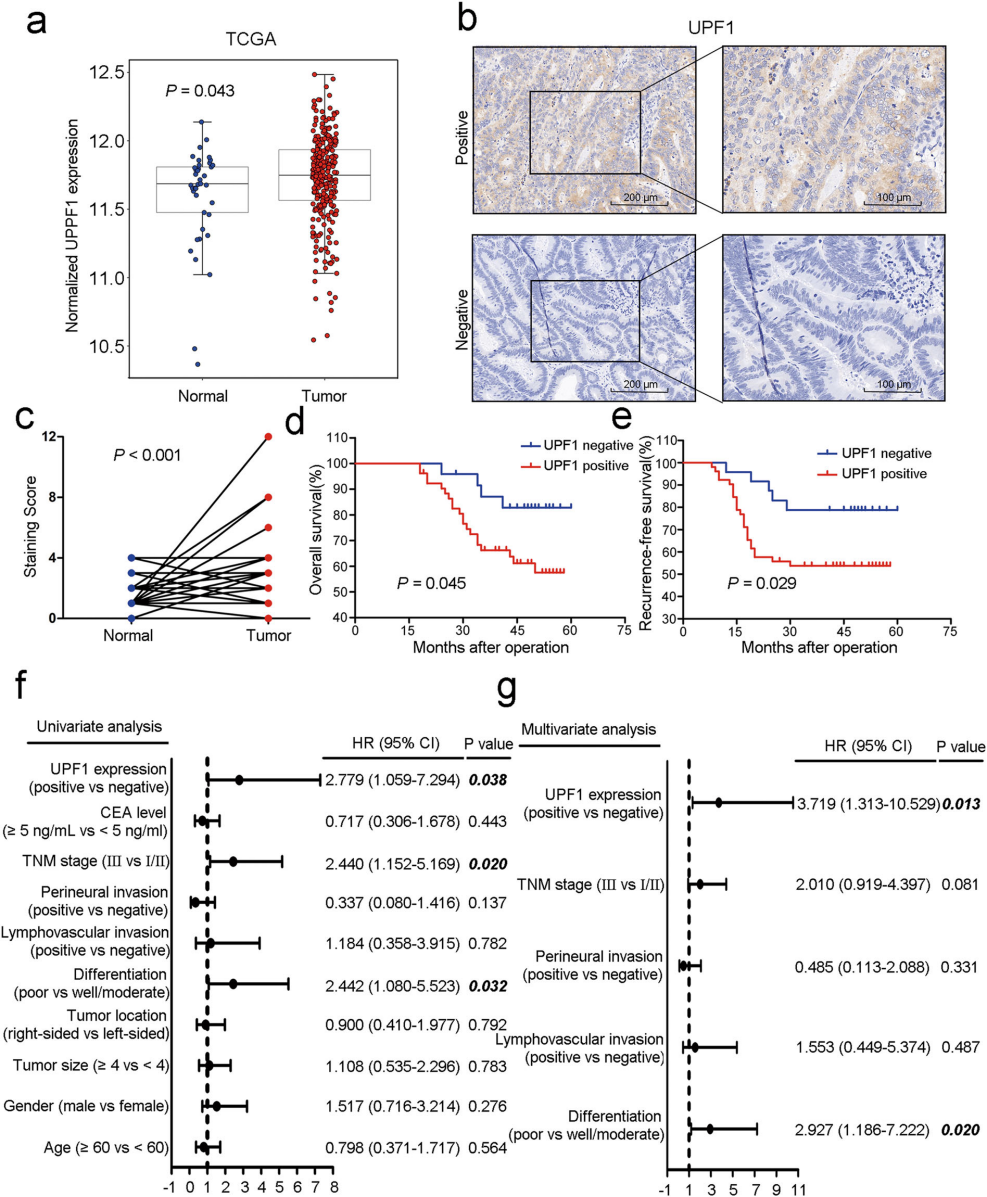
UPF1 is upregulated in CRC and predicted a poor prognosis. **a** UPF1 was overexpressed in CRC tissues in mRNA level from TCGA datasets. **b** Images of UPF1 in IHC staining in CRC tissue microarrays, scale bar, 200 μm and 100 μm. **c** UPF1 was overexpressed in CRC tissues in protein level by IHC staining in tissue microarrays. **d**–**e** UPF1 positive predicted worse OS and RFS in CRC patients. **f**–**g** Univariate and multivariate COX regression analysis revealed that UPF1 was an independent risk factor for recurrence in CRC patients.

To investigate the expression of UPF1 in CRC tissues, we performed bioinformatics analysis of UPF1 in mRNA level using the public RNA sequencing datasets from The Cancer Genome Atlas (TCGA)^22^ and found that UPF1 was overexpressed (*P* = 0.043, Fig. 1a). We also detected it by IHC staining of tissue microarrays using a retrospective cohort containing 76 patients with paired CRC tumor and normal tissues. The representative IHC images of UPF1 staining were shown in Fig. 1b. Similarly, the results verified that UPF1 was dramatically upregulated in CRC tissues (*P* < 0.001, Fig. 1c). In these patients, UPF1-positive was detected in 52 (68.4 %) of the tumor tissues, whereas only 4 (5.3 %) of the normal tissues were UPF1-positive (Table 1, *P* < 0.001).

**Table. 1:** Clinicopathological characteristics of CRC patients according to UPF1 or recurrence status.

	All cases (*n* = 76)	UPF1 negative	UPF1 positive	*P* value	Non-recurrence	recurrence	*P* value
Tissue				***0.000***			
Normal	76	72	4				
Tumor	76	24	52				
Age				0.916			0.380
<60	45 (59.2%)	14 (58.3%)	31 (59.6%)		26 (55.3%)	19 (65.5%)	
≥60	31 (40.8%)	10 (41.7%)	21 (40.4%)		21 (44.7%)	10 (34.5%)	
Gender				0.639			0.265
Female	35 (46.1%)	12 (50.0%)	23 (44.2%)		24 (51.1%)	11 (37.9%)	
Male	41 (53.9%)	12 (50.0%)	29 (55.8%)		23 (48.9%)	18 (62.1%)	
Tumor size				0.145			0.760
<4 cm	41 (53.9%)	10 (41.7%)	31 (59.6%)		26 (55.3%)	15 (51.7%)	
≥4 cm	35 (46.1%)	14 (58.3%)	21 (40.4%)		21 (44.7%)	14 (48.3%)	
Tumor location				0.444			0.299
Left-sided	29 (38.2%)	9 (37.5%)	20 (38.5%)		15 (31.9%)	14 (48.3%)	
Right-sided	25 (32.9%)	10 (41.7%)	15 (28.8%)		16 (34.0%)	9 (31.0%)	
Rectum	22 (28.9%)	5 (20.8%)	17 (32.7%)		16 (34.0%)	6 (20.7%)	
Differentiation				0.067			0.059
Well/moderate	64 (84.2%)	17 (70.8%)	47 (90.4%)		43 (91.5%)	21 (72.4%)	
Poor	12 (15.8%)	7 (29.2%)	5 (9.6%)		4 (8.5%)	8 (27.6%)	
Lymphovascular invasion				0.983			1.000
Negative	68 (89.5%)	22 (91.7%)	46 (88.5%)		42 (89.2%)	26 (89.7%)	
Positive	8 (10.5%)	2 (8.3%)	6 (11.5%)		5 (10.6%)	3 (10.3%)	
Perineural invasion				0.631			0.178
Negative	64 (84.2%)	19 (79.2%)	45 (86.5%)		37 (78.7%)	27 (93.1%)	
Positive	12 (15.8%)	5 (20.8%)	7 (13.5%)		10 (21.3%)	2 (6.9%)	
TNM stage				0.625			***0.040***
I	7 (9.2%)	2 (8.3%)	5 (9.6%)		6 (12.8%)	1 (3.4%)	
II	35 (46.1%)	13 (54.2%)	22 (42.3%)		25 (53.2%)	10 (34.5%)	
III	34 (44.7%)	9 (37.5%)	25 (48.1%)		16 (34.0%)	18 (62.1%)	
CEA level				0.692			0.361
<5 ng/mL	53 (69.7%)	16 (66.7%)	37 (71.2%)		31 (66.0%)	22 (75.9%)	
≥5 ng/mL	23 (30.3%)	8 (33.3%)	15 (28.8%)		16 (34.0%)	7 (24.1%)	
TOP2A expression				***0.012***			0.530
Negative	18 (23.7%)	10 (41.7%)	8 (15.4%)		10 (21.3%)	8 (27.6%)	
Positive	58 (76.3%)	14 (58.3%)	44 (84.6%)		37 (78.7%)	21 (72.4%)	
UPF1 expression							***0.035***
Negative	24 (31.6%)				19 (40.4%)	5 (17.2%)	
Positive	52 (68.4%)				28 (59.6%)	24 (82.8%)	

*P* value in **bold** and *italic* indicates significant.

To explore the clinical significance of UPF1 in CRC, we analyzed the correlation between the expression of UPF1 and clinicopathological characteristics of CRC patients (Table 1). A significant association was observed between UPF1 positive and TOP2A positive (*P* = 0.012, 58.3% in UPF1-negative group vs 84.6% in UPF1-positive group). High UPF1 expression (*P* = 0.035) and high TNM stage (*P* = 0.040) more likely result in recurrence in CRC patients. However, high expression of UPF1 and recurrence in CRC patients had no correlation with gender, age, tumor location, tumor size, lymphovascular invasion, perineural invasion, and carcinoembryonic antigen level. Additionally, Kaplan–Meier curves showed a strong correlation between UPF1 higher expression and a poorer OS (*P* = 0.045, Fig. 1d). UPF1-positive patients also had a shorter recurrence-free survival (RFS) (*P* = 0.029, Fig. 1e). In addition, univariate and multivariate COX regression analysis suggested that high expression of UPF1 (HR = 3.719; 95% CI = 1.313 to 10.529; *P* = 0.013) and poor differentiation (HR = 2.927; 95% CI = 1.186 to 7.222; *P* = 0.020) were independent prognostic risk factors for recurrence in CRC (Fig. 1f, g). In summary, UPF1 is aberrantly upregulated in CRC and high expression of UPF1 predicts a poor prognosis in CRC patients.

### UPF1 promotes oxaliplatin resistance in CRC in vitro

**Fig. 2:**
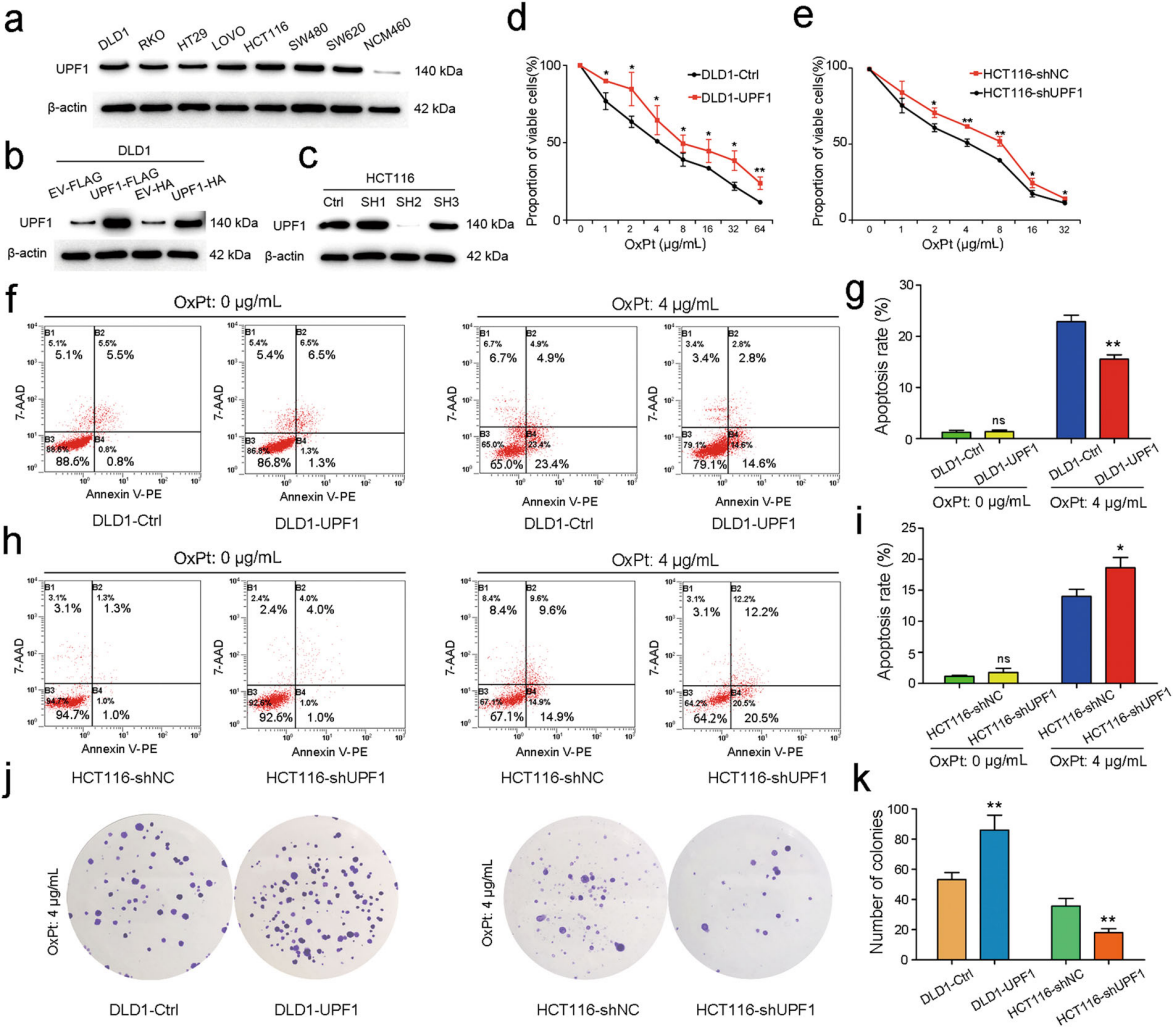
UPF1 induced chemoresistance to oxaliplatin (OxPt) in CRC in vitro. **a** The baseline expression of UPF1 in CRC cell lines and normal colonic epithelial cell line detected by Western blot. **b**–**c** Overexpression and knockdown of UPF1 in DLD1 and HCT116, respectively. **d**–**e** Cell viability of stable CRC cell lines treated with a gradient concentration of oxaliplatin assessed by CCK-8 assay. **f**–**i** Apoptosis rate of stable CRC cell lines treated with or without oxaliplatin assessed by flow cytometry. **j**–**k** Clone formation of stable CRC cell lines treated with oxaliplatin.

The baseline expression of UPF1 in CRC cell lines and normal colonic epithelial cell line was detected by immunoblot. Notably, UPF1 expression in the normal cell line, NCM460, was remarkably less compared with CRC cell lines (Fig. 2a). We chose DLD1 to overexpress UPF1 due to its relative low baseline expression. And based on the same rule, UPF1 was knocked down in HCT116 using three small hairpin RNAs (shRNAs). Overexpression and knockdown were confirmed by Western blotting and SH2 was the most efficient in UPF1 knockdown (Fig. 2b, c). HA and FLAG tags were used for co-immunoprecipitation (co-IP) assay. Drug cytotoxicity assay showed that overexpression of UPF1 decreased oxaliplatin sensitivity in DLD1 while knockdown of UPF1 increased oxaliplatin sensitivity in HCT116 (Fig. 2d, e). In apoptosis assay, overexpression and knockdown of UPF1 had no influence on apoptosis rate in DLD1 and HCT116, respectively. Whereas after treatment for 48 h, oxaliplatin-induced apoptosis was significantly reduced in DLD1-UPF1 and raised in HCT116-shUPF1 (Fig. 2f–i). Clone formation assay also affirmed that UPF1 promoted oxaliplatin resistance in CRC in vitro. The number of colonies were more in DLD1-UPF1 group and less in HCT116- shUPF1 group compared with control group with treatment of oxaliplatin for 48 h (Fig. 2j, k). However, the size of colonies remained no obvious change between different groups, which indicated that UPF1 may have no effect on cell proliferation. CCK-8 assay also showed that UPF1 did not influence proliferation in DLD1 and HCT116 cell lines (Fig. S1a, b).

### UPF1 promotes oxaliplatin resistance in CRC in vivo

**Fig. 3:**
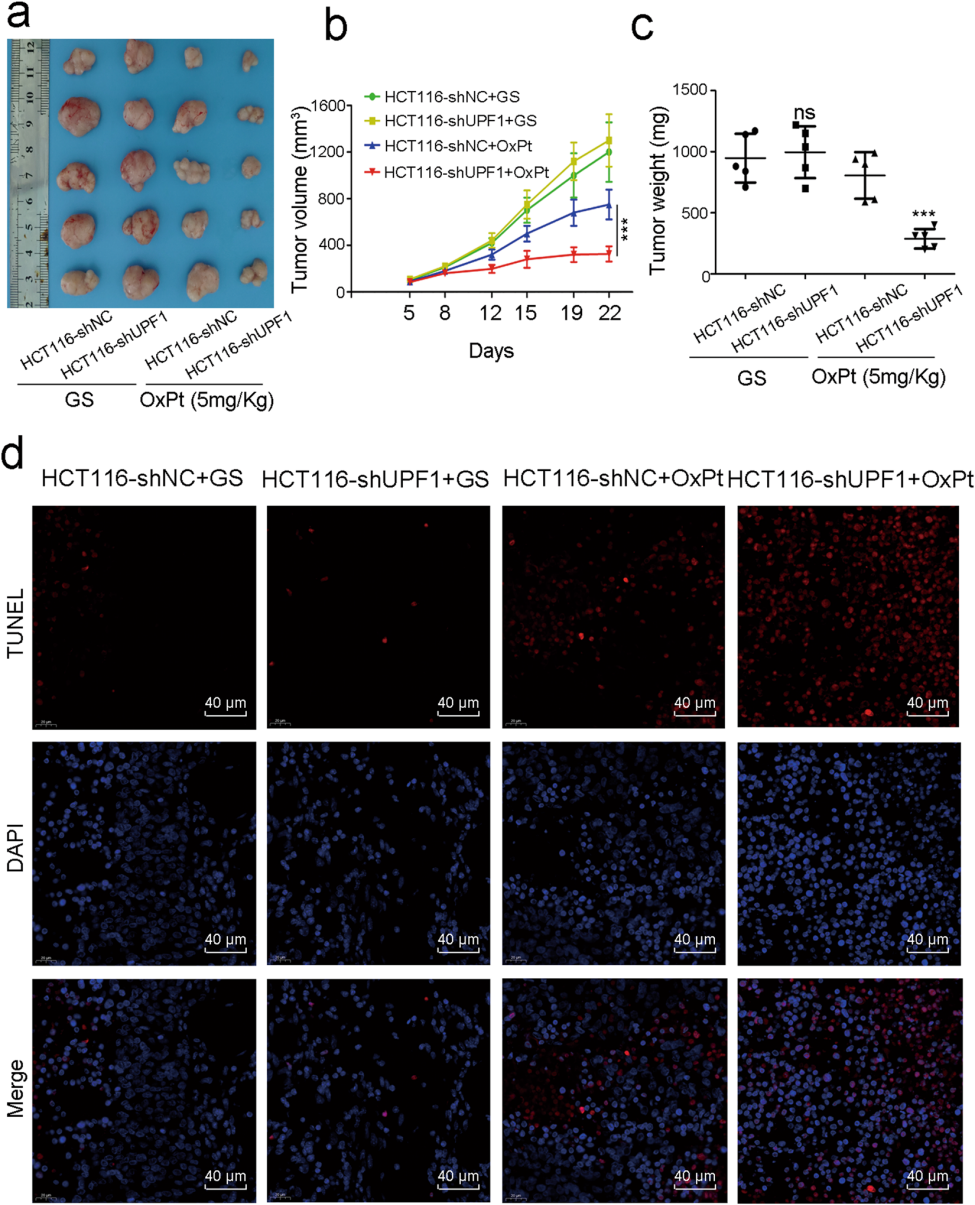
UPF1 promoted chemoresistance to oxaliplatin in CRC in vivo. **a** Xenografts of HCT116-shNC and HCT116-shUPF1 treated with or without oxaliplatin. **b** Tumor volume in nude mice bearing HCT116 cells in different groups. Tumor volume equaled length × width^2^ × 0.5 and was measured twice a week. **c** Tumor weight in nude mice bearing HCT116 cells in different groups. **d** TUNEL assay was performed to detect tumor cell apoptosis in xenograft tumor tissues, scale bar, 40 μm.

Nude mice were divided into 2 groups and injected with HCT116-shNC and HCT116-shUPF1 cell lines, respectively. One half of each group was treated with 5% glucose solution and the other treated with oxaliplatin (5 mg/kg). The tumor xenografts were shown in Fig. 3a. In the groups treated with glucose solution, there is no significant difference between HCT116-shNC and HCT116-shUPF1 groups. Whereas, xenografts in HCT116-shNC group grew faster and were much larger and heavier after treatment with oxaliplatin (Fig. 3b, c). In line, these findings were further confirmed by TUNEL assay (Fig. 3d). Apoptosis rate in tissues remained no change in HCT116-shUPF1 but after being treated with oxaliplatin, apoptosis rate remarkably raised compared with control. Xenografts assay in vivo revealed that UPF1 promotes oxaliplatin resistance in CRC.

### UPF1-induced oxaliplatin resistance is involved with interaction with TOP2A and increase of phosphorylated TOP2A in a SMG1-dependent manner

**Fig. 4:**
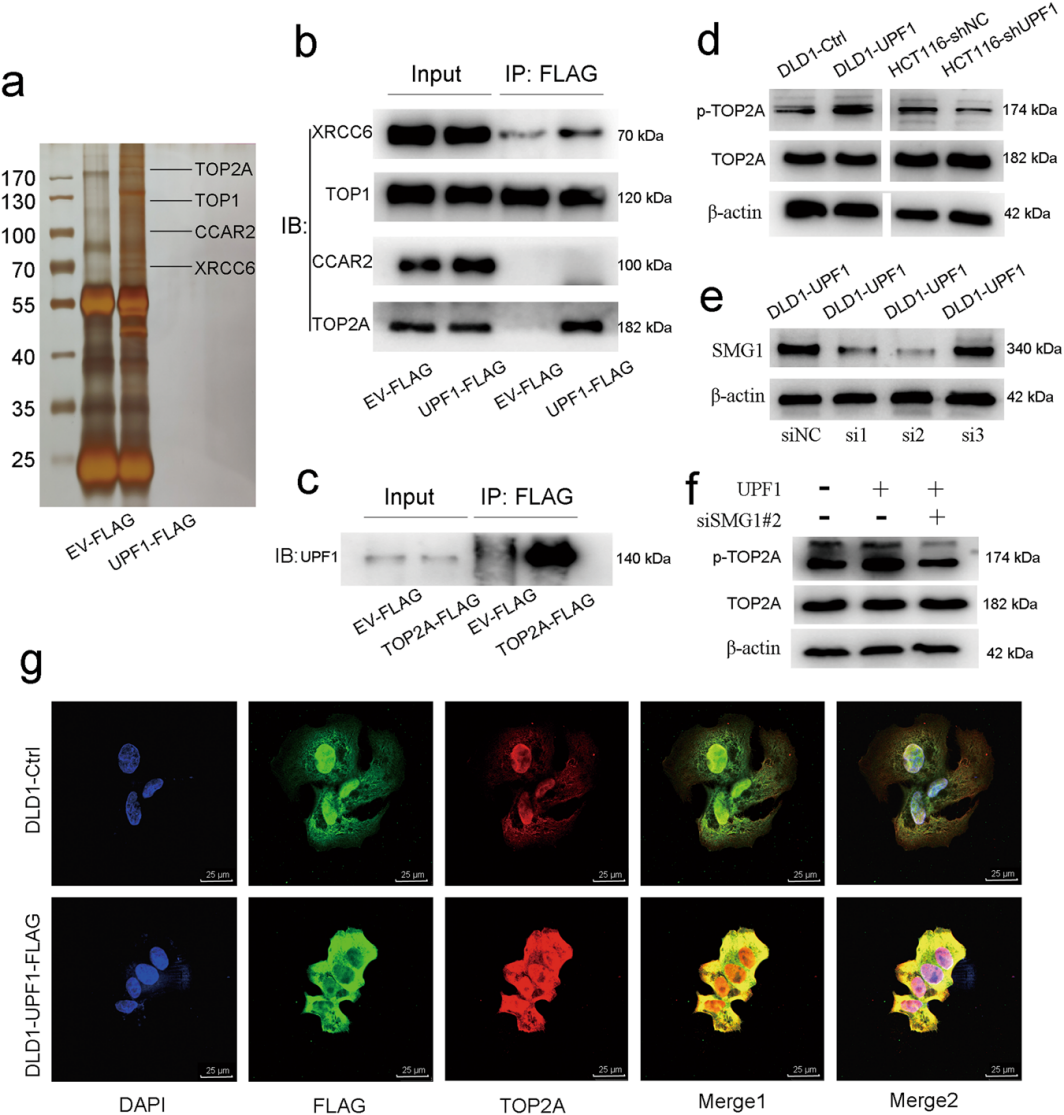
UPF1 interacted with TOP2A and increased phosphorylated TOP2A. **a** Signature bands of DLD1-UPF1 cell line emerged after silver staining. The location of TOP2A, TOP1, CCAR2, and XRCC6 were marked. **b**–**c** Interaction of UPF1 and TOP2A proved in co-IP assay. (**d**) UPF1 had no influence on the total expression of TOP2A but raised phosphorylated TOP2A at the site of Ser^1106^. **e** SMG1 was silenced using small hairpin RNAs. **f** UPF1 raised phosphorylated TOP2A and the elevation was brought to a halt by knockdown of SMG1. **g** Images of immunocytochemistry assay showed interaction between UPF1 and TOP2A, scale bar, 25 μm.

167 kinds of proteins were identified by silver staining (Fig. 4a) and mass spectrometry (MS) which were listed in additional file S1. MS score represents the reliability of detected proteins to a certain extent. UPF1 was identified with the highest MS score (3992.20). Known interacting partners of UPF1, such as UPF2 (MS score = 36.92) and SMG1 (MS score = 0.00)^23^ were also identified. TOP2A (MS score = 132.22), TOP1 (MS score = 61.59), CCAR2 (MS score = 53.75) and XRCC6 (MS score = 95.64) were selected as candidates for UPF1 interacted proteins according to pathway analysis and biological functions. TOP2A was proved to be upregulated in mRNA and protein levels in CRC tissues (Fig. S1c–e). Enrichment analysis exhibited related pathways and TOP2A was unearthed to be involved in platinum-resistant pathway (Fig. S1f). The co-IP assay showed the interaction between UPF1 and TOP2A, but not TOP1, CCAR2 and XRCC6 (Fig. 4b). Similarly, UPF1 was also precipitated using an anti-FLAG resin in HEK-293T cell line transfected into TOP2A-FLAG (Fig. 4c). We further found that expression of total TOP2A remained no significant change but phosphorylated TOP2A at the site of Ser^1106^ was increased after upregulation of UPF1. Correspondingly, silencing UPF1 had no influence on total TOP2A expression but attenuated phosphorylated TOP2A at the same site (Fig. 4d). UPF1 shows protein phosphokinase activity neither in published papers nor in its biological functions. SMG1, a member of the PI3K (phosphoinositide 3-kinase related kinases) family, is also a key factor in NMD. SMG1 could interact with UPF1 and directly phosphorylates UPF1^24–26^. In our study, SMG1 was also identified in mass spectrometry (additional file S1). Co-IP assay and ICC demonstrated that SMG1 could interact with UPF1 (Fig. S2). Phosphorylated TOP2A was elevated in DLD-UPF1 compared with control. By knockdown of SMG1 in DLD1-UPF1, elevation of phosphorylated TOP2A was brought to a halt (Fig. 4e, f). SMG1 may play a role in UPF1-induced phosphorylation of TOP2A. In DLD1 cell line transfected into UPF1-FLAG, the results of immunocytochemistry indicated the colocalization of UPF1 and TOP2A (Fig. 4g).

**Fig. 5:**
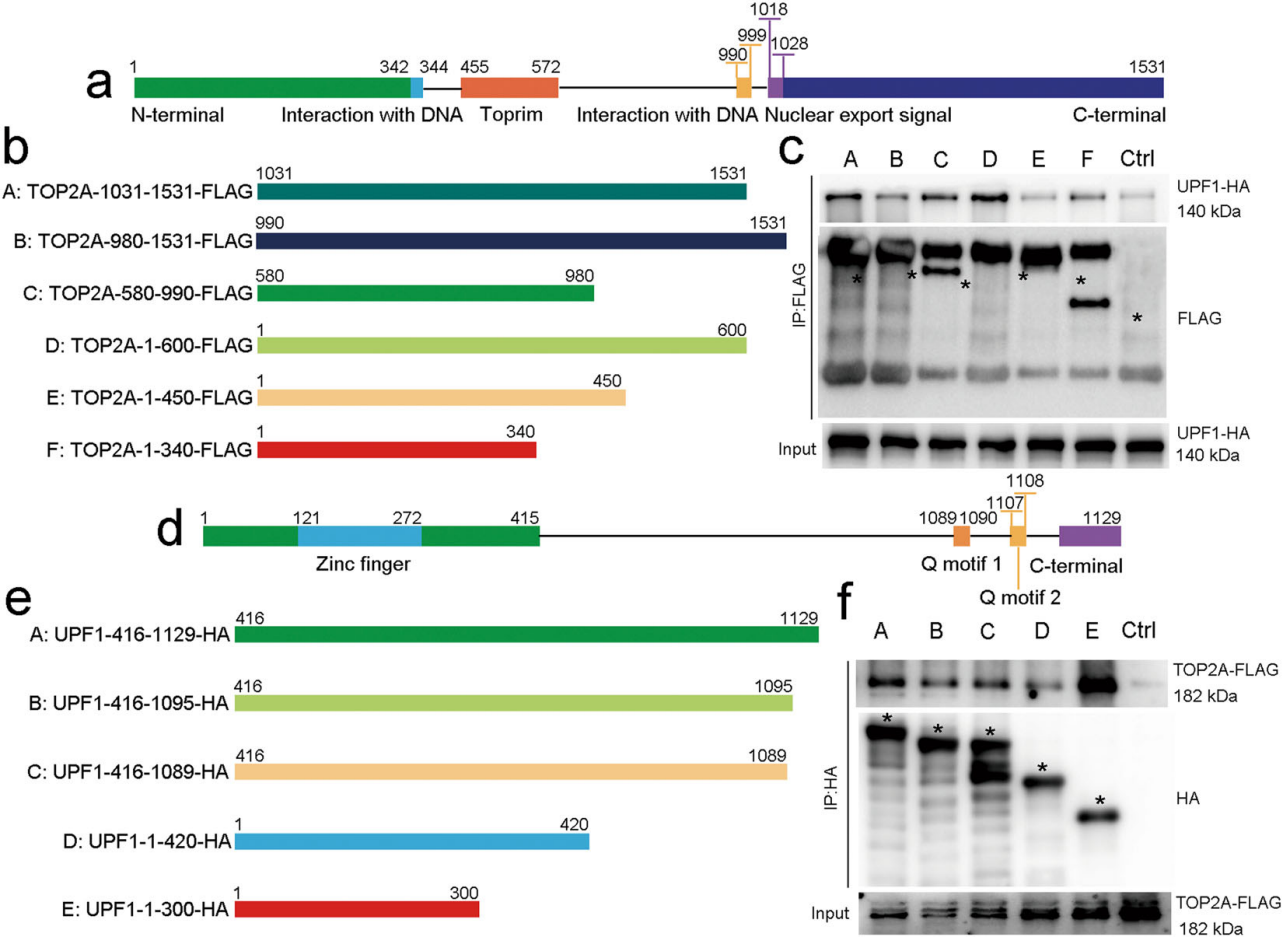
Co-IP assays using truncated mutants identified the precise binding region between TOP2A and UPF1. **a** Schematic diagram of key domains in TOP2A. **b** Six truncated mutants of TOP2A fused with FLAG tag were generated according to its key domains. **c** Co-IP assays were performed of the lysates from HEK-293T cells transfected with HA-tagged UPF1 and FLAG-tagged truncated mutants of TOP2A. **d** Schematic diagram of key domains in UPF1. **e** Five truncated mutants of UPF1 fused with HA tag were generated according to its key domains. **f** Co-IP assays were performed of the lysates from HEK-293T cells transfected with FLAG-tagged TOP2A and HA-tagged truncated mutants of UPF1.

To identify the binding region between UPF1 and TOP2A, we generated the truncated mutants of UPF1 and TOP2A to perform co-IP assays. The full length of TOP2A contains 1531 amino acids and six truncated mutants were generated (Fig. 5a, b). Co-IP assay revealed that Toprim domain may be the binding region with UPF1 (Fig. 5c). The full length of UPF1 contains 1129 amino acids and five truncated mutants were generated (Fig. 5d, e). The results elucidated that the zinc finger domain of UPF1 may interact with TOP2A (Fig. 5f).

**Fig. 6:**
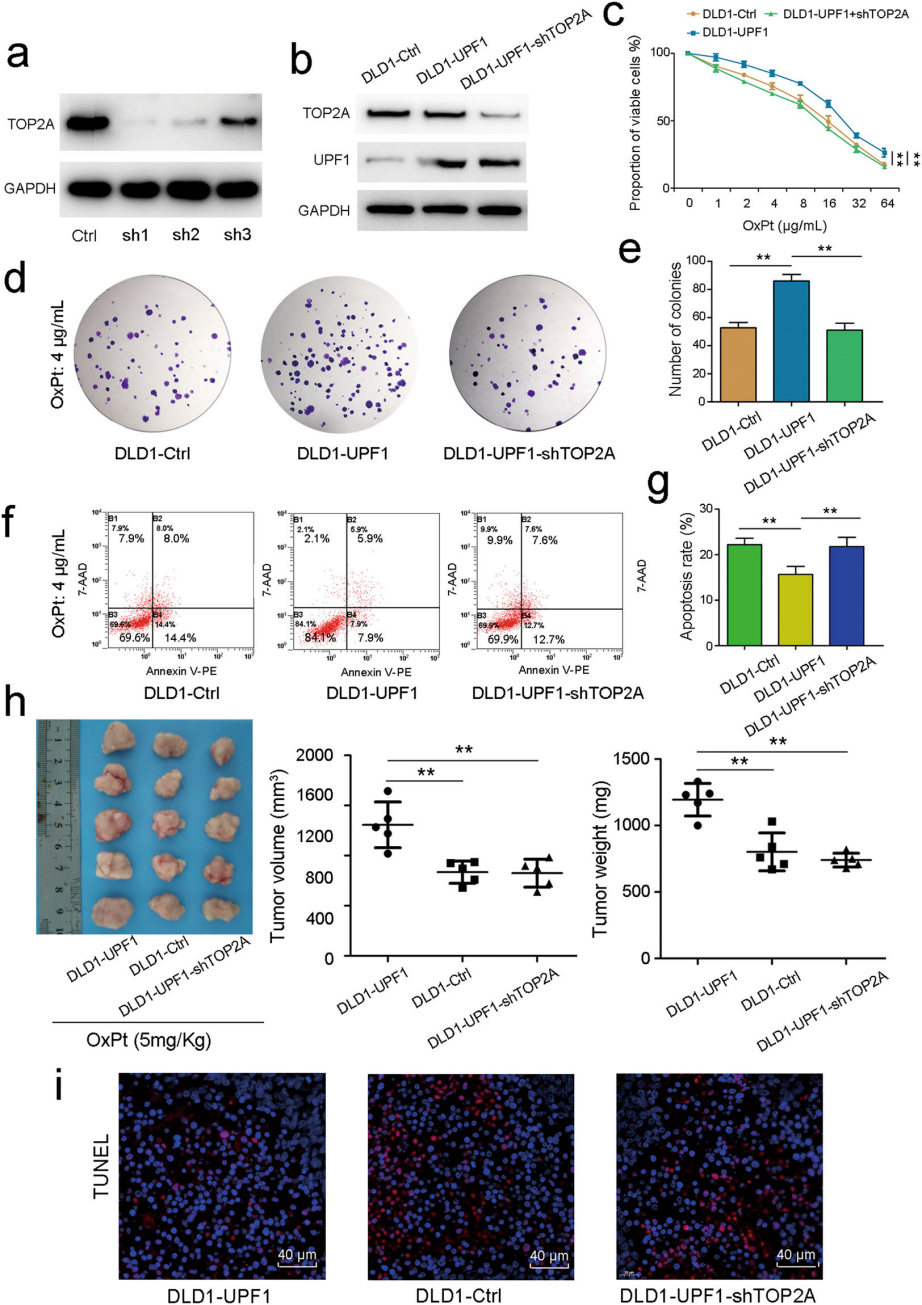
UPF1-induced oxaliplatin resistance was abolished by silencing the TOP2A gene in vitro and in vivo. **a**, **b** TOP2A was silenced using small hairpin RNAs. **c** Drug cytotoxicity assay, **d**, **e** clone formation assay, and **f**, **g** flow cytometry showed abrogation of chemoresistance to oxaliplatin induced by UPF1 by knockdown of TOP2A. **h** Xenografts of DLD1-UPF1, DLD1-Ctrl, and DLD1-UPF1-shTOP2A treated with oxaliplatin and tumor volume and weight of xenografts in different groups. **i** TUNEL assay was performed to detect tumor cell apoptosis in xenograft tumor tissues, scale bar, 40 μm.

To verify the function of TOP2A in UPF1-induced oxaliplatin resistance, TOP2A was silenced using three small hairpin RNAs. Sh1 was selected for following experiments due to its biggest efficiency (Fig. 6a, b). Of note, drug cytotoxicity assay demonstrated that UPF1-induced resistant cells regained the sensitivity to oxaliplatin after knockdown of TOP2A (Fig. 6c). As shown in clone formation assay, DLD1-Ctrl, DLD1-UPF1, and DLD1-UPF1-shTOP2A were treated by oxaliplatin for 48 h. We can see that UPF1 overexpression significantly increased the number of colonies while the effect was abolished by silencing the TOP2A gene in the cells. The size of colonies had no difference among different groups (Fig. 6d, e). The results in apoptosis assay came to the same conclusion (Fig. 6f, g). In vivo, xenografts in DLD1-UPF1 group were much larger and heavier under treatment with oxaliplatin. The trend of chemoresistance was brought to a halt with TOP2A knockdown (Fig. 6h). In same, TUNEL assay testified that inhibition of apoptosis in tissue induced by UPF1 was deactivated by silencing TOP2A (Fig. 6i). Altogether, these data strongly suggested that TOP2A played an essential role in the UPF1-induced chemoresistance to oxaliplatin in CRC.

### UPF1 maintains stemness in CRC in a TOP2A-dependent manner

**Fig. 7:**
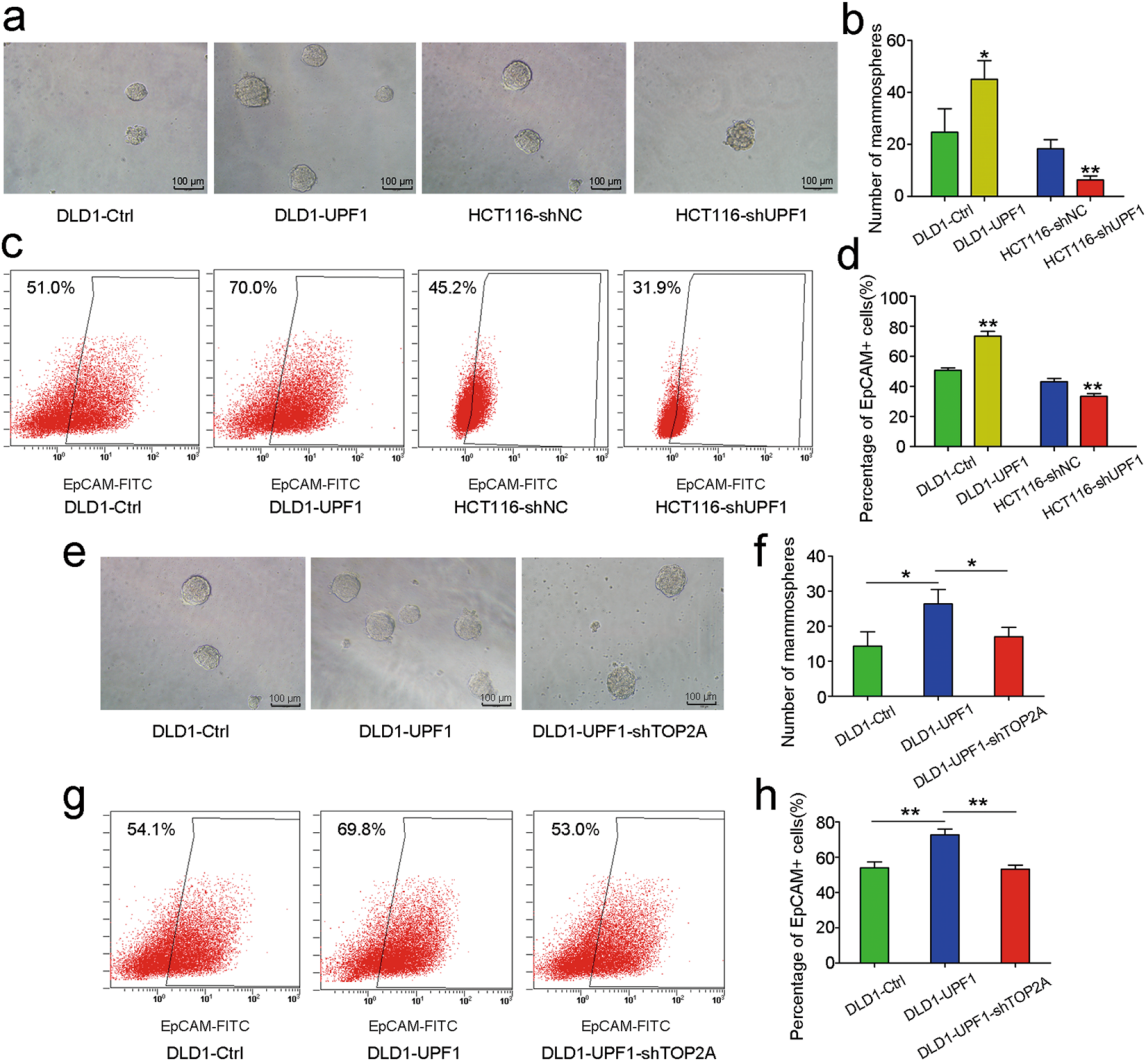
UPF1 maintains stemness in CRC in a TOP2A-dependent manner. **a**–**b** The ability of mammosphere formation was enhanced after upregulation of UPF1 in DLD1 and was attenuated after knockdown of UPF1 in HCT116, scale bar, 100 μm. **c**, **d** The percentage of EpCAM-positive cells was raised in DLD1-UPF1 and diminished in HCT116-shUPF1 compared with control. **e**–**f** Silencing TOP2A abrogated enhancement of the ability of mammosphere formation induced by UPF1, scale bar, 100 μm. **g**, **h** Silencing TOP2A down regulated the rise of the percentage of EpCAM-positive cells induced by UPF1.

The ability of mammosphere formation was enhanced after upregulation of UPF1 in DLD1 and was attenuated after knockdown of UPF1 in HCT116 (Fig. 7a, b). EpCAM has been proposed as one of the cancer stem cell (CSC) markers^27–29^. In our study, the percentage of EpCAM-positive cells was raised in DLD1-UPF1 and diminished in HCT116-shUPF1 (Fig. 7c, d). Notably, UPF1-induced enhancement of stemness was abated by silencing the expression of TOP2A in mammosphere formation assay (Fig. 7e, f). Meanwhile, UPF1-induced change of EpCAM+ cell rates was abolished by knockdown of TOP2A (Fig. 7g, h).

## Discussion

CRC maintains a high rate of incidence and mortality worldwide, as well as that in China^1,2^. Stage II CRC exhibits a recurrence rate of about 20% after radical resection^30^. For patients with stage III disease, recurrence rate can be as high as more than 50%^31^. Chemotherapy resistance is a major cause of recurrence and poor prognosis in CRC patients^32^.

Oxaliplatin, as a DNA interacting agent, results in DNA damage and induction of DNA strand breaks thus activates apoptosis signal pathway in cancer cells^33^. Oxaliplatin is the first platinum drug with proven activity against colorectal tumors. Unfortunately, drug resistance brings failure to treatment, which becomes a pressing problem in clinical treatment in CRC patients.

It is reported that UPF1 can play a role in regulated mRNA and protein decay^34^. In addition, UPF1 is proved to dysregulate in multiple tumors and influence carcinogenesis^13–15^. However, there is lack of detailed study of UPF1 in CRC. It is reported that UPF1 might play a part in the selection of target gene mutations with a functional role in MSI-H carcinogenesis^35^. In line, Ada Collura et al. ascertained that inhibition of nonsense mediated decay in vivo using amlexanox reduced MSI tumor growth^36^. Currently, the function of UPF1 and potential mechanisms remain unclear in CRC.

Firstly, we demonstrated that UPF1 was aberrantly overexpressed in CRC tissues. And high UPF1 expression more likely resulted in recurrence and predicted a worse OS in CRC patients. In addition, UPF1-positive was an independent prognostic risk factor for recurrence in CRC.

Herein, how UPF1 acts as a oncogene in CRC needed deep study. CCK−8, clone formation assays and flow cytometry indicated that UPF1 may have no effect on cell proliferation and apoptosis. While assays in vitro showed that overexpression of UPF1 weakened sensitivity to oxaliplatin in DLD1 while knockdown of UPF1 reinforced sensitivity to oxaliplatin in HCT116. In vivo, we reached the same conclusion that UPF1 promotes oxaliplatin resistance in CRC.

Afterwards, the underlying mechanism of UPF1-induced oxaliplatin resistance remained to be revealed. Mass spectrometry analysis identified 166 kinds of proteins that may interact with UPF1. Enrichment analysis exhibited related pathways and TOP2A was unearthed to be involved in platinum-resistant pathway. Kyoto Encyclopedia of Genes and Genomes pathway map showed the detailed signal network in platinum drug resistance (map01524)^37^. Oxaliplatin-induced DNA-adducts activated nucleotide excision repair through Topoisomerase II, which could attenuate DNA damage and result in oxaliplatin resistance^38^. TOP2A, DNA topoisomerase, is an enzyme that controls and alters the topologic states of DNA during transcription^39^. TOP2A is upregulated and could induce tumor development and progression in multiple tumors^40–43^ and is proved important therapeutic target of anticancer agents^44^. Etoposide, as an inhibitor of TOP2A, is a kind of cell cycle specific antitumor drug and applied in multiple tumors. The catalytic activity of TOP2A can be modulated by interactions with various proteins^44^. It is reported that phosphorylation of serine^1106^ in the catalytic domain of TOP2A regulates enzymatic activity and drug sensitivity^45,46^. In CRC tissues, TOP2A was proved to be upregulated in mRNA and protein levels. In the IHC staining of tissue microarrays, a significant association was observed between UPF1 positive and TOP2A positive. TOP1 could modulate colorectal cancer response to irinotecan^47^. CCAR2 overexpression decreased the chemosensitivity to oxaliplatin in CRC^48^. XRCC6, as a DNA repair gene, may participate in platinum resistance by modulating the DNA repair capacity^49^. TOP2A, TOP1, CCAR2, and XRCC6 were selected as candidates for UPF1 interacted proteins according to pathway analysis and biological functions. Forward and reverse co-IP assays proved the interaction of UPF1 with TOP2A, instead of TOP1, CCAR2, and XRCC6. In DLD1 transfected into UPF1-FLAG, the results of immunocytochemistry assay also indicated the colocalization of UPF1 and TOP2A. To identify the binding region between UPF1 and TOP2A, we generated the truncated mutants of UPF1 and TOP2A to perform co-IP assays. Toprim in TOP2A is a catalytic domain involved in DNA strand breakage and rejoining and may be the binding region with UPF1. The zinc fingers vary widely in structure, as well as in function. And zinc finger domain of UPF1 may interact with Toprim in TOP2A. YacG is a bacterial type chromosome-encoded and zinc-finger containing protein. A crystal structure of the YacG-DNA gyrase complex revealed that the interaction between zinc-finger of YacG with the Toprim domain regulated TOP2A activity^50,51^. In our study, interaction with UPF1 may modulate the enzyme activity of TOP2A in a similar manner. We further found that expression of total TOP2A remained no significant change after upregulation of UPF1. While the interaction with UPF1 increased phosphorylated TOP2A at the site of Ser^1106^. However, UPF1 shows protein phosphokinase activity neither in published papers nor in its biological functions. The mechanism of phosphorylation of TOP2A remains unknown. SMG1, a member of the PI3K (phosphoinositide 3-kinase related kinases) family, is also a key factor in NMD. SMG1 could interact with UPF1 and directly phosphorylate UPF1. The formation of SMG1-UPF1-eRF1-eRF3 complex (SURF) to the exon junction complex triggers UPF1 phosphorylation and NMD^24–26^. In our study, SMG1 was also identified in mass spectrometry. Co-IP assay and ICC demonstrated that SMG1 could interact with UPF1. Phosphorylated TOP2A was elevated in DLD-UPF1 compared with control. By knockdown of SMG1 in DLD1-UPF1, elevation of phosphorylated TOP2A was brought to a halt. SMG1 may play a role in UPF1-induced phosphorylation of TOP2A. Of note, UPF1-mediated oxaliplatin resistance was abolished by silencing the TOP2A gene in vitro and in vivo.

Cancer stem cells (CSCs) are defined by their functional properties and could be able to self-renew and propagate the tumor^52^. CSC-like properties or stemness have an essential role in the drug resistance in CRC^53,54^. In our study, the stemness was progressed in UPF1-upregulation cell line and was impeded in UPF1-knockdown cell line. CSCs are characterized by specific markers, such as EpCAM. Flow cytometry indicated that the percentage of EpCAM-positive cells was diminished after silencing UPF1 and increased after upregulating UPF1. TOP2A is abundant in pluripotent embryonic stem cells and inhibition of TOP2A noticeably decreases pluripotency and differentiation potential. TOP2A-inactivated embryonic stem cells failed to generate embryonic bodies and thus do not differentiate^55^. Thus, we concluded that TOP2A could regulate stemness in a certain manner. Notably, UPF1-induced CSC-like properties was abated by silencing the expression of TOP2A. Altogether, these data strongly suggested that TOP2A played an essential role in the UPF1-induced chemoresistance of CRC cells in response to oxaliplatin in vitro and in vivo and TOP2A could be a therapeutic target in UPF1-overexpressed CRC patients.

There are some limitations in this study that could be addressed in future research. We have not figured out how interaction between UPF1 and TOP2A changes the enzyme activity. And we wonder whether combination with the drugs targeted for TOP2A could make synergistic effect in patients with highly expressed UPF1.

## Conclusions

**Fig. 8:**
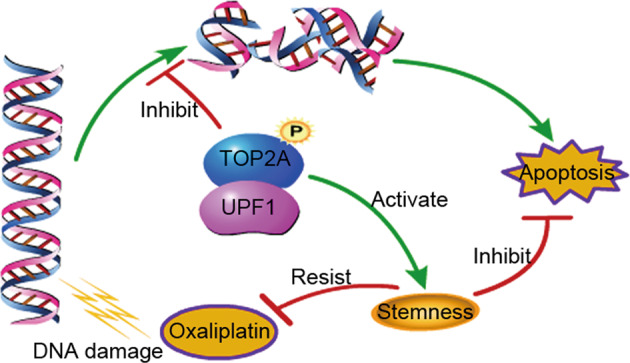
Schematic diagram of this study. UPF1 protects CRC cells from oxaliplatin-induced apoptosis via regulation of TOP2A activity and maintenance of stemness.

UPF1 was overexpressed and predicted a poor prognosis in CRC. UPF1 enhanced chemoresistance to oxaliplatin in CRC, which may result from regulation of TOP2A activity and maintenance of stemness. Our findings could provide a new therapy strategy for chemoresistance to oxaliplatin in CRC patients (Fig. 8).

## Materials and methods

### Patients and specimens

76 patients were pathologically diagnosed with CRC and received surgical therapy at Fudan University Shanghai Cancer Center. Patients who received neoadjuvant radiochemotherapy were excluded from our study. Specimens from the 76 patients were made into tissue microarrays. Our study was carried out in accordance with the requirements of the Biomedical Ethics Committee of Fudan University Shanghai Cancer Center.

### Cell lines and culture

CRC cell lines (DLD1, HT29, LOVO, HCT116, RKO, SW480, SW620) and HEK−293T cell line were purchased from Type Culture Collection Cell Bank, Chinese Academy of Sciences. Normal colonic epithelial cell line (NCM460) was purchased from American Type Culture Collection. All cell lines were cultured in Dulbecco’s Modified Eagles Medium with 10% fetal bovine serum, penicillin (10^7^ U/L) and streptomycin (10 mg/L) and incubated at 37 °C in a humidified atmosphere containing 5% CO_2_ in Fudan University Shanghai Cancer Center.

### Lentivirus production and transfection

Core plasmids of UPF1 were purchased from OBiO Technology (Shanghai, China) and core plasmids of TOP2A were purchased from Genechem (Shanghai, China). The detailed information of core plasmids was shown in additional file S2. Core plasmids were co-transfected with psPAX2 and pMD2.G into HEK−293T cells using Hieff Trans^TM^ Liposomal Transfection Reagent (Yeasen, Shanghai, China). 48 h later, virus supernatant was collected. 3 × 10^5^ cells were cultured in a 6-well plate. After incubation in virus supernatant for 48 h, stable cell lines were selected with puromycin or flow cytometry and transfected efficiency was evaluated by immunoblot.

### Transient transfection

To construct truncated mutants of UPF1 and TOP2A, specific primers (additional file S3) were designed to subclone corresponding sequences into pLVX-IRES-Puro vector. Small interfering RNAs (siRNAs) of SMG1 were purchased from Biomics Biotech (Jiangsu, China). The sequences of siRNA were shown in additional file S2. Plasmids overexpressing UPF1-HA and FLAG-tagged TOP2A truncated mutants or overexpressing TOP2A-FLAG and HA-tagged UPF1 truncated mutants were co-transfected into HEK-293T cells using Hieff Trans^TM^ Liposomal Transfection Reagent (Yeasen, Shanghai, China). 48 h later, cells were lysed for co-immunoprecipitation (Co-IP) assay. siRNAs were transfected into cells using Lipofectamine 3000 Transfection Reagent (Invitrogen, Carlsbad, USA). 48 h later, cells were lysed for Western blotting.

### Flow cytometry

Apoptosis assay was conducted using Annexin V-PE/7-AAD apoptosis detection kit (Yeasen, Shanghai, China). EpCAM-positive cells were detected using FITC-conjugated EpCAM antibody (Sino Biological, Beijing, China). Briefly, 3 × 10^5^ cells were cultured in a 6-well plate with or without being treated with oxaliplatin for 48 h. Adherent cells and cells in supernatant were collected and washed twice. Cells suspended with 1× binding buffer with the addition of 5 μL Annexin V and 10 μL 7-AAD or 10 μL EpCAM antibody. After incubation protecting from light for 15 min, apoptosis rate or EpCAM-positive cells were detected by Cytomics FC 500 MPL Flow cytometer (Beckman, USA).

### Western blotting

Total protein was extracted with NuPAGE^®^ LDS sample buffer (Thermo Fisher Scientific, Waltham, USA). A certain amount of protein was separated by SDS-PAGE gel and transferred to PVDF membranes. After blockage with non-fat milk powder and incubation with primary antibodies and horseradish peroxidase-conjugated secondary antibodies, images were captured with ImageQuant™ biomolecular imager (General Electric, USA). All the antibodies used in our study were listed in additional file S4.

### Drug cytotoxicity assay

Drug cytotoxicity was evaluated by Cell Counting Kit-8 (CCK-8) assay. Cell suspension was seeded in a 96-well plate and cells were treated with a gradient concentration of oxaliplatin for 48 h after adhesion. Medium containing CCK-8 reagent (Yeasen, Shanghai, China) was added to each well of the plate. After two-hours’ incubation, absorbance at 450 nm was detected by microplate reader (BioTek, USA).

### Clone formation assay

1000 cells were cultured in a 6-well plate and treated with oxaliplatin for 48 h after adhesion. The medium was replaced by the fresh every three days. 2 weeks later, cells were fixed with 4% paraformaldehyde for 30 min and stained with crystal violet for 30 min. Images were photographed and visible colonies were counted.

### Co-immunoprecipitation (Co-IP) assays, silver staining, and mass spectrometry

Cells in culture dish were lysed with IP buffer containing protease inhibitors and phosphatase inhibitor cocktails (Topscience, Shanghai, China). IP buffer was a mixture of 1 mM EDTA, 20 mM HEPES, 150 mM NaCl, 0.05% sodium deoxycholate, and 0.05% NP-40. Lysate was spun at the speed of 12,000 rpm at 4 °C for 12 min. 60 μL was kept for input and the rest of the extract was immunoprecipitated with anti-HA beads or anti-FLAG resins at 4 °C for more than 3 h. After washing 5 times with IP buffer, beads or resins of protein bound were lysed with 1.25× SDS loading buffer and boiled at 100 °C for 10 min. Silver staining was conducted according to the manufacture of Fast Silver Stain Kit (Beyotime, Shanghai, China). Bands appeared after electrophoresis and silver staining in SDS-PAGE gel received mass spectrometry analysis (Wayen Biotechnologies, Shanghai, China) for protein identification. Interacted protein was further confirmed by immunoblot.

### Mammosphere formation assay

Mammosphere formation assay was used to define stemness in vitro in our study and conducted as previously described^56^. Cells were digested by trypsin and washed twice. 300 cells suspended in 200 μL mammosphere medium were seeded in a 96-well plate with ultra-low attachment surface. The formulation of mammosphere medium was shown in additional file S5. 10 days later, images were photographed with an Olympus microscope system and mammospheres were counted.

### Immunohistochemistry (IHC)

IHC was performed as previously described^57^. The results of IHC staining were determined by immunoreactive score, ranging from 0 to 12. Immunoreactive score equals the number of positive cells multiplied by the staining intensity. The number of cells stained were divided into 5 groups: 0, 1–10%, 11–50%, 51–80%, and 81–100%, corresponding to 0–4 score, respectively. And staining intensity has 4 levels, negative, weak, moderate, and strong, corresponding to 0–3 score, respectively.

### Immunocytochemistry

Sterilized round coverslips were laid in a 24-well plate and cells were seeded on them. After 24 h incubation, cells were fixed with 4% paraformaldehyde for 30 min and permeabilized on ice with 0.25% Triton X−100. Then cells were blocked with bovine serum albumin and incubated with primary antibodies for 2 h and fluorescent secondary antibodies protecting from light for 1 h at room temperature. After mounting with DAPI Fluoromount mounting medium for 15 min, images were captured using Leica confocal system.

### Xenograft experiments

5 × 10^6^ cells were subcutaneously injected into four-week-old male BALB/c nude mice. 5 days later, mice were randomly divided into groups and treated with oxaliplatin (5 mg/kg) or 5% glucose solution by intraperitoneal injection, twice per week for three weeks, respectively. All mice were sacrificed and tumors were collected and weighed. Tumor volume equaled length × width^2^ × 0.5 and was measured twice a week. Xenografts were saved in 4% paraformaldehyde for following experiments. Apoptosis rates in the xenograft were determined by terminal deoxynucleotidyl transferase-mediated dUTP nick end labeling (TUNEL) assay using TMR (red) TUNEL Cell Apoptosis Detection Kit (Servicebio, Wuhan, China). Xenograft experiments were approved by the Committee on Animals Handling of Fudan University Shanghai Cancer Center.

### Statistical analysis

Data are shown in mean ± standard deviation. All analyses were performed by IBM SPSS 22.0 software. Quantitative variables were analyzed using Student’s *t* test. The Log-rank test in the Kaplan–Meier method and the COX regression model were used to assess patients’ survival outcome and prognostic factors. All the experiments were performed in triplicate. A two-tailed value of *P* < 0.05 was considered statistically significant (*P < 0.05; **P < 0.01; ***P < 0.001).

### Ethics approval and consent to participate

Our study was carried out in accordance with the requirements of the Biomedical Ethics Committee of Fudan University Shanghai Cancer Center. Informed consent was obtained from all patients included in the study. Xenograft experiments were approved by the Committee on Animals Handling of Fudan University Shanghai Cancer Center.

## Supplementary information

S1

S2

S3

S4

S5

Supplementary figure legends

Figure S1

Figure S2

## Data Availability

The data used to support the findings of this study are included within the article and the supplementary materials.
