# Supplementary material for: UPF1 promotes chemoresistance to oxaliplatin through regulation of TOP2A activity and maintenance of stemness in colorectal cancer
Source: Cell Death Dis. 2021 May 21;12(6):519. doi: 10.1038/s41419-021-03798-2 (PMC8140095; doi:10.1038/s41419-021-03798-2)
Supplement: Supplementary file 2 — S2 [file 41419_2021_3798_MOESM2_ESM.docx]

| UPF1-FLAG | pRLenti-CMV-UPF1-3FLAG-PGK-Puro |
| --- | --- |
| UPF1-HA | pRLenti-CMV-UPF1-HA-PGK-Puro |
| TOP2A-FLAG | CMV-TOP2A-3FLAG-SV40-Neomycin |

shRNA sequences of UPF1

| **NO.** | **5’** | **STEM** | **Loop** | **STEM** | **3’** |
| --- | --- | --- | --- | --- | --- |
| Y9274-F | Ccgg | GCTCGCAGACTCTCACTTT | cTCAAGAGA | AAAGTGAGAGTCTGCGAGC | TTTTTTg |
| Y9274-R | aattcaaaaaa | GCTCGCAGACTCTCACTTT | TCTCTTGAg | AAAGTGAGAGTCTGCGAGC |  |
| Y9275-F | Ccgg | CCACCTGCTGAACTACTAT | TTCAAGAGA | ATAGTAGTTCAGCAGGTGG | TTTTTTg |
| Y9275-R | aattcaaaaaa | CCACCTGCTGAACTACTAT | TCTCTTGAA | ATAGTAGTTCAGCAGGTGG |  |
| Y9276-F | Ccgg | GCCTCATGCAGTTCAGCAA | TTCAAGAGA | TTGCTGAACTGCATGAGGC | TTTTTTg |
| Y9276-R | aattcaaaaaa | GCCTCATGCAGTTCAGCAA | TCTCTTGAA | TTGCTGAACTGCATGAGGC |  |

shRNA sequences of TOP2A

| **ID** | **5’** | **STEM** | **Loop** | **STEM** | **3’** |
| --- | --- | --- | --- | --- | --- |
| TOP2A-RNAi(18395-1)-a | Ccgg | gcCCAAGTGTTCTTTAGCTTT | CTCGAG | AAAGCTAAAGAACACTTGGGC | TTTTTg |
| TOP2A-RNAi(18395-1)-b | aattcaaaaa | gcCCAAGTGTTCTTTAGCTTT | CTCGAG | AAAGCTAAAGAACACTTGGGC |  |
| TOP2A-RNAi(18396-1)-a | Ccgg | gcCTGATTTGTCTAAGTTTAA | CTCGAG | TTAAACTTAGACAAATCAGGC | TTTTTg |
| TOP2A-RNAi(18396-1)-b | aattcaaaaa | gcCTGATTTGTCTAAGTTTAA | CTCGAG | TTAAACTTAGACAAATCAGGC |  |
| TOP2A-RNAi(18397-1)-a | Ccgg | gcTCCAAATCAATATGTGATT | CTCGAG | AATCACATATTGATTTGGAGC | TTTTTg |
| TOP2A-RNAi(18397-1)-b | aattcaaaaa | gcTCCAAATCAATATGTGATT | CTCGAG | AATCACATATTGATTTGGAGC |  |

siRNA sequence of SMG1

| **ID** | **Sequences (5’-3’)** | |
| --- | --- | --- |
| hs-SMG1-si-1 | Sense | CCGUCCUAGUGAACUUUAUdTdT |
|  | Antisense | AUAAAGUUCACUAGGACGGdTdT |
| hs-SMG1-si-2 | Sense | GAGACUGCCUAUAAGUUAAdTdT |
|  | Antisense | UUAACUUAUAGGCAGUCUCdTdT |
| hs-SMG1-si-3 | Sense | GCGGCGUUAUUUGAACUAAdTdT |
|  | Antisense | UUAGUUCAAAUAACGCCGCdTdT |
