# Supplementary material for: UPF1 promotes chemoresistance to oxaliplatin through regulation of TOP2A activity and maintenance of stemness in colorectal cancer
Source: Cell Death Dis. 2021 May 21;12(6):519. doi: 10.1038/s41419-021-03798-2 (PMC8140095; doi:10.1038/s41419-021-03798-2)
Supplement: Supplementary file 4 — S4 [file 41419_2021_3798_MOESM4_ESM.docx]

Antibodies used in our research

| Name | Manufacturers | Catalog No. | Applications |
| --- | --- | --- | --- |
| UPF1 | Abcam | ab109363 | WB, IHC |
| TOP2A | Abclonal Technology | A16440 | WB |
| TOP2A | Servicebio | GB111293 | IHC, ICC |
| CCAR2 | Abclonal Technology | A7126 | WB |
| XRCC6 | Abclonal Technology | A0883 | WB |
| TOP1 | Abclonal Technology | A12524 | WB |
| GAPDH | Proteintech | 60004-1-Ig | WB, |
| β-actin | Abclonal Technology | AC028 | WB |
| Goat anti-Rabbit Secondary Antibody | Proteintech | SA00001-2 | WB |
| Goat anti-Mouse Secondary Antibody | Proteintech | SA00001-1 | WB |
| FLAG | MBL | M185-3L | WB, ICC |
| HA | Cell signaling technology | 3724 | WB |
| EpCAM | Sino Biological | 10694-MM06-F | FCM |
| p-TOP2A | Abcam | ab75765 | WB |
| p-TOP2A | Abcam | ab52853 | WB |
| Goat anti-Mouse IgG (H+L) Cross-Adsorbed Secondary Antibody, Alexa Fluor 488 | Invitrogen | A11001 | ICC |
| Goat anti-Rabbit IgG (H+L) Cross-Adsorbed Secondary Antibody, Alexa Fluor 555 | Invitrogen | A21428 | ICC |
| SMG1 | Abcam | ab30916 | WB, ICC |
| Goat anti-Rabbit IgG (H+L) Cross-Adsorbed Secondary Antibody, Alexa Fluor 488 | Invitrogen | A11008 | ICC |
| Goat anti-Mouse IgG (H+L) Cross-Adsorbed Secondary Antibody, Alexa Fluor 555 | Invitrogen | A21422 | ICC |
