# Supplementary material for: UPF1 promotes chemoresistance to oxaliplatin through regulation of TOP2A activity and maintenance of stemness in colorectal cancer
Source: Cell Death Dis. 2021 May 21;12(6):519. doi: 10.1038/s41419-021-03798-2 (PMC8140095; doi:10.1038/s41419-021-03798-2)
Supplement: Supplementary file 5 — S5 [file 41419_2021_3798_MOESM5_ESM.docx]

The formulation of mammosphere medium

| Component | Initial concentration | Final concentration | Volume in 50mL |
| --- | --- | --- | --- |
| DMEM | - | - | 24 mL |
| DMEM/F12(1:1) | - | - | 24 mL |
| BSA/PBS | 10% | 0.4% | 2 mL |
| B27 | 50× | 0.2× | 200 μL |
| EGF | 50mg/mL | 10ng/mL | 1 μL |
| FGF | 50mg/mL | 10ng/mL | 1 μL |
