# Supplementary material for: UPF1 promotes chemoresistance to oxaliplatin through regulation of TOP2A activity and maintenance of stemness in colorectal cancer
Source: Cell Death Dis. 2021 May 21;12(6):519. doi: 10.1038/s41419-021-03798-2 (PMC8140095; doi:10.1038/s41419-021-03798-2)
Supplement: Supplementary file 6 — Supplementary figure legends [file 41419_2021_3798_MOESM6_ESM.docx]

**Fig. S1** (**a-b**) UPF1 had no influence on proliferation in DLD1 and HCT116 cell lines. **(c)** TOP2A was overexpressed in CRC tissues in mRNA level from TCGA datasets. (**d**) Images of TOP2A in IHC staining in CRC tissue microarray, scale bar, 150 μm and 50 μm. **(e)** TOP2A was overexpressed in CRC tissues in protein levels by IHC staining in tissue microarrays. (**f**) Enrichment analysis of proteins identified in mass spectrometry located TOP2A in platinum drug resistance.

**Fig. S2 (a)** Interaction of UPF1 and SMG1 proved in co-IP assay. **(b)** Images of immunocytochemistry assay showed interaction between UPF1 and SMG1, scale bar, 25 μm.
