# Supplementary figures and images for: UPF1 promotes chemoresistance to oxaliplatin through regulation of TOP2A activity and maintenance of stemness in colorectal cancer
Source: Cell Death Dis. 2021 May 21;12(6):519. doi: 10.1038/s41419-021-03798-2 (PMC8140095; doi:10.1038/s41419-021-03798-2)

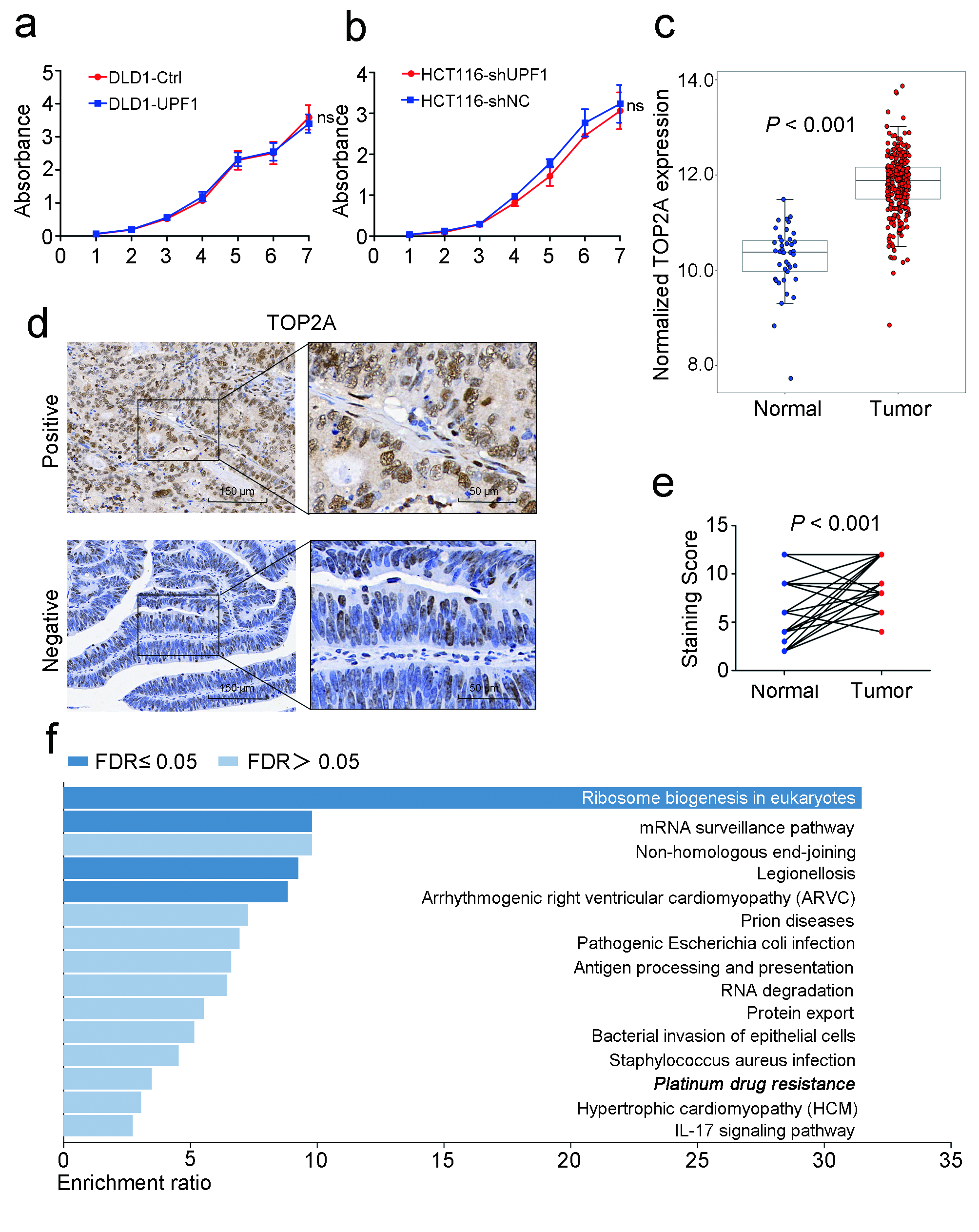

Supplement: Supplementary file 7 — Figure S1 [file 41419_2021_3798_MOESM7_ESM.tif]

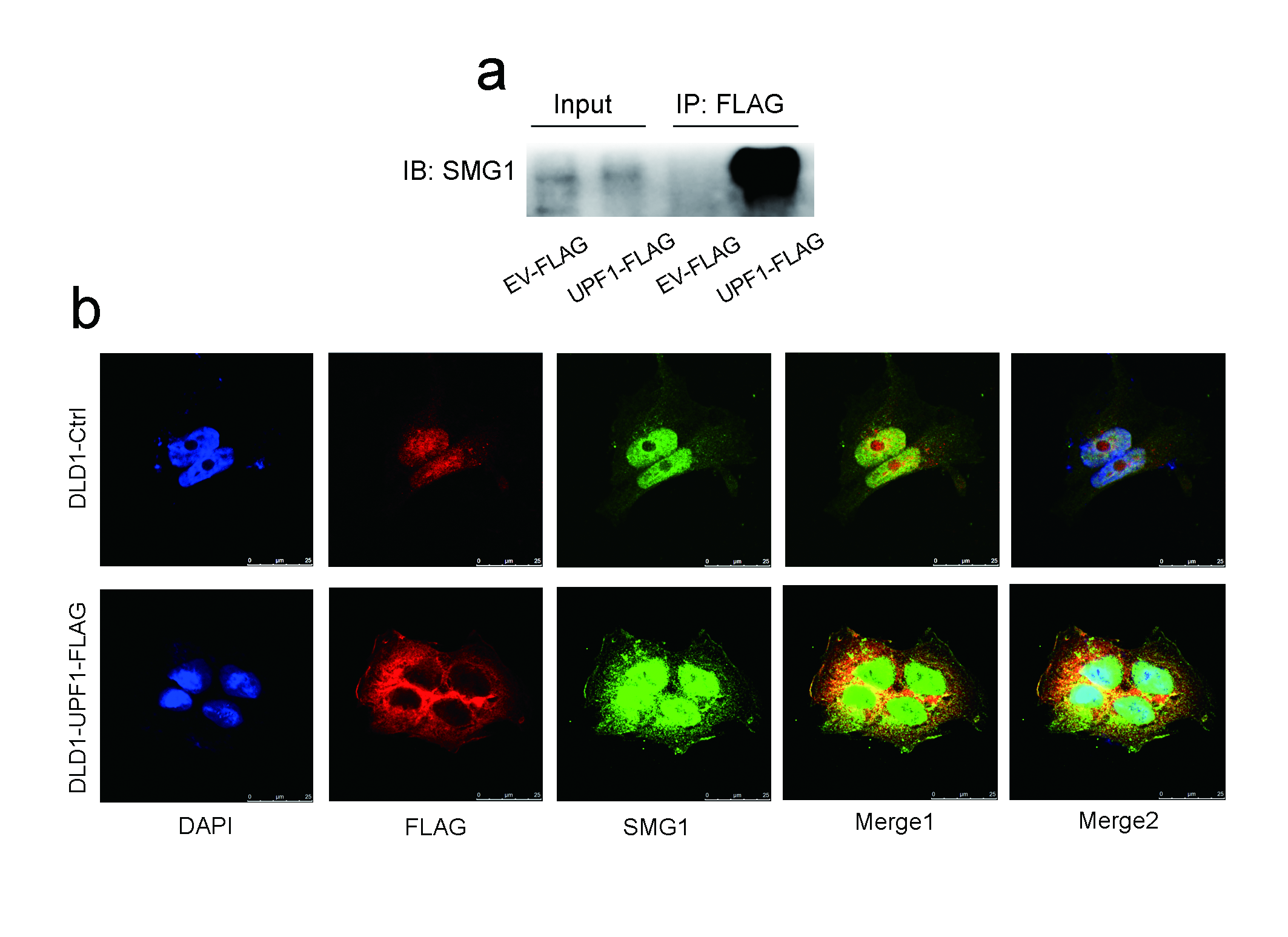

Supplement: Supplementary file 8 — Figure S2 [file 41419_2021_3798_MOESM8_ESM.tif]
